# The Use of Alternative Rooms in Forensic and Regular Psychiatric Units: A Scoping Review

**DOI:** 10.3390/healthcare11172432

**Published:** 2023-08-31

**Authors:** Alexandre Hudon, Maria Alexandra Rosca, Olivier La Charité-Harbec, Jeanne-Marie Allard, Stéphanie Borduas Pagé

**Affiliations:** 1 Centre de Recherche de l’Institut Universitaire en Santé Mentale de Montréal, Montreal, QC H1N 3V2, Canada; 2 Department of Psychiatry and Addictology, Faculty of Medicine, Université de Montréal, Montreal, QC H3T 1J4, Canada; maria.alexandra.rosca@umontreal.ca; 3 Institut Universitaire en Santé Mentale de Montréal, Montreal, QC H1N 3M5, Canada; olivier.la.charite-harbec.cemtl@ssss.gouv.qc.ca (O.L.C.-H.); jeanne-marie.allard.cemtl@ssss.gouv.qc.ca (J.-M.A.)

**Keywords:** acute psychiatry, alternative rooms, emotion regulation, violence, psychiatric units

## Abstract

(1) Background: Emotional regulation, distress and relational conflicts often occur during hospitalization and rehabilitation on psychiatric units, especially in patients suffering from severe and persistent mental disorders. While widely used in children and geriatric patients, little literature exists on the use and outcomes of alternative rooms in the context of forensic and regular psychiatric units for adult patients. Considering the scarcity of the literature on alternative use, this study is motivated by the following research question: what are the main uses and outcomes of alternative rooms in the context of forensic and regular psychiatric units? The main objective of this study is to conduct a scoping review of the use and outcomes of alternative rooms for the context of psychiatric inpatients. (2) Methods: A systematic search was performed in the electronic databases of MedLine, Web of Science, PsycNet (PsycINFO) and Google Scholar from their inception dates until 2022. (3) Results: A total of nine studies were analyzed. Sensory, multisensory rooms, Snoezelen, and comfort rooms are the types of alternative rooms discussed in these studies. Distress and anxiety reduction, increase in self-esteem, impact on seclusion rates, patient–staff communication and alliances, heart and respiration rate reduction, and improvement of alexithymia were identified among the main uses and outcomes of these rooms. (4) Conclusions: The scarcity of literature available to draw information from for this review and possible impact on improving patient outcomes and quality of treatment in psychiatric units opens the door to future studies to better understand the efficacy of such rooms. Research into the ideal implementation tactics of such rooms, long-term outcomes, and the influence on diverse patient demographics could be areas of improvement in the use of alternative rooms.

## 1. Introduction

### 1.1. Challenges on Psychiatric Wards

Patients hospitalized on psychiatric units can experience difficulties related to emotional regulation, stress management, and relational conflicts with their peers and the staff. This is especially the case for patients suffering with persistent and severe mental disorders. Inpatient stays are often linked to improvement in emotional regulation and interpersonal regulation of emotions in patients with severe mental disorders [1]. A large study that included 2683 psychiatric inpatients ranging from 18 to 81 years, demonstrated an association between emotional regulation and suicide attempts, highlighting the need for additional studies in this population [2]. A recent review of the literature identified 18 publications on patients’ experiences and factors that evoked emotional reactions during hospitalization and confirmed that hospitalization increases patient’s emotions and feelings of depression and anxiety [3]. Another recent review of the literature showed that various factors, such as having a diagnosis of schizophrenia combined with substance abuse, lengthy hospitalization, and situational factors, can yield violent behaviors that have been identified in previous studies [4]. Workplace conflicts linked to these difficulties and physical assaults occurring on psychiatric units can bring about emotional and psychological consequences on the staff working on them which negatively impacts patient care [5]. To improve patient care and reduce the frequency of these incidents, several studies reported the importance of psychiatric ward design to reduce aggressive behaviors and improve the experience of patients on such units [6]. However, a recent literature review on psychiatric ward design, reports that there is no strong causal link between the design of psychiatric units and patients’ clinical outcomes, thus warranting further study [7]. While this might be the case, the therapeutic and legal contexts of such wards can influence their design. In forensic psychiatry, the design of psychiatric units is of utmost importance as it needs to address security issues (patients and staff). It also needs to provide a secure environment to build effective therapeutic relationships, while also considering that impulsivity, emotion regulation, and violence experienced by the patients are often frequent challenges that are encountered [8].

### 1.2. Risk Management and Approaches

While there are no specific guidelines to systematically address emotion regulation, violent behaviors, and relational conflicts experienced by patients in an inpatient psychiatric context, various approaches are available. For example, psychopharmacological approaches can be used when violent behavior poses a threat to the patient or the public (e.g., staff or other patients) [9]. Chemical restraints, while sometimes effective, are temporary, and are bound to potential negative side effects (adverse events) such as extreme sedation, respiratory depression, extrapyramidal symptoms, and hypotension [10]. Other medications, such as antagonisms of dopaminergic receptors (e.g., quetiapine, olanzapine) can be used for anxiety and impulsivity; often administered on an off-label basis [11]. The effectiveness of such approaches has been questioned by authors of several studies considering the associated greater risk of adverse events [12]. Non-psychopharmacological methods can also be used. Depending on the context, the type of intervention will vary. For example, for aggressivity and violent behaviors, seclusion, manual (or mechanical) restraints, and de-escalation can be used [13]. However, restraints and seclusive approaches can raise several ethical concerns and may contribute to the burden of care in the context of psychiatric inpatient units [14]. Physical restraints and other seclusion techniques, as an example, pose significant adverse events as they can cause additional burden for the patients and the medical workers [15]. This could augment difficulties in emotional regulation and violent behaviors.

One interesting non-pharmacological approach is the use of alternative rooms (also known as secure rooms or comfort rooms) [16,17]. There is no clear definition for such rooms. In the adult population, alternative rooms can be used as therapeutic options for patients with a various array of medical difficulties as well as a mean for emotional regulation. As an example, a study on comfort room indicates the need to find alternative approaches to the use of seclusion and restraints such as these rooms to account for negative outcomes linked to usual aspects of behavioral controlling techniques [18]. However, they often present different objects or means to stimulate the patient to exteriorize their emotions. This is to be distinguished from seclusion rooms in which patients are often deprived of sensory stimulants. Patients use alternative rooms on a voluntary basis. Another distinction while comparing seclusions rooms and alternative rooms is that seclusion rooms are often negatively perceived by patients as they remind them of prison cells and can evoke traumatic experiences [19]. In the children and adolescent population, the use of alternative rooms is vastly discussed.

### 1.3. Sensory, Multisensory and Alternative Rooms

While there is a vast body of literature on the use of such rooms for children, adolescents and the geriatric population, very little research exists for adult psychiatric inpatients. Studies on multisensory rooms, such as Snoezelen rooms, demonstrated various positive outcomes for children and adolescents. For example, in children suffering from autism disorder, the use of such a room showed a decrease in stereotyped speech [20]. Another study, by Bobier et al. (2015), reported the use of a sensory room for children and adolescents to reduce seclusion rates [21]. In the geriatric population, a recent scoping review reported the use of sensory approaches or rooms as having a potential positive impact on the quality of life of senior residents [22]. As of today, to our knowledge, there is no study that encompasses the different uses of alternative rooms and their effects on patients in psychiatric contexts. We employ the name alternative room in this study to encompass all rooms that use sensory modalities on psychiatric inpatient units as they are named heterogeneously in the literature.

### 1.4. Research Question, Objectives and Hypothesis

Considering the scarcity of literature on alternative use, the research question driving this study is as follows: what are the main uses and outcomes of alternative rooms in the context of forensic and regular psychiatric units? The main objective of this scoping review is to identify the main uses and outcomes of alternative rooms in the context of forensic and regular psychiatric units. We hypothesized that there are various usages of these rooms, and they can help stabilize emotional distress, reduce violent behaviors, and improve therapeutic relationships between the health providers and the patients. The usage of such a room might be effective in reducing violent behaviors, improve emotional regulation and solidify patient–staff relationships. This study could provide further understanding on this non-psychopharmacological avenue to approach a broad array of difficulties encountered by the patients during their stay in a psychiatric ward.

## 2. Materials and Methods

### 2.1. Search Strategies

A systematic search was performed in the electronic databases of EMBASE, MEDLINE, APA and CINAHL from their inception dates until 2022. Text words and indexing terms (MeSH) with keywords inclusive for the fields of alternative rooms (e.g., room, sensory room, sports, relaxation, gym, ludic, quiet, recreative, etc.) and psychiatric units (e.g., mental hospital, psychiatric services, hospital, ward, center, department) were used. These inclusive terms were selected as they best comprise the use of different types of rooms in the context of psychiatric services. The study’s design was influenced by the Preferred Reporting Items for Systematic Reviews and Meta-Analyses (PRISMA) statement. The main electronic search strategy that was conducted is available in Appendix A. The conceptualization and implementation of the search methodology were conducted with the help of an experienced librarian specialized in mental health at the Institut universitaire en santé mentale de Montréal (IUSMM). There were no setting or geographical restrictions that were applied. The search strategy performed was limited to English or French language sources. Duplicates were removed with the help of Clarivate’s Endnote software solution.

### 2.2. Study Eligibility

Studies were included if they met the following criteria: (1) the study is about (or the use of) a type of physical room that acts as a non-medical approach to patients suffering from any psychiatric illness; (2) the study was conducted in the field of psychiatry, notably mental health; (3) the use of this room is in an adult inpatient context and either in a forensic psychiatric unit or a regular psychiatric unit; (4) the patients using the rooms are between 18 and 65 years of age as they encompass most definitions of the adult population. Considering there is currently no consensus as to what constitutes an alternative room, any room which uses a non-medical (e.g., medication) approach to help the patient was included in the presented study. Unpublished articles and studies were not included. Studies published in another language than English or French were excluded to avoid interpretation biases.

### 2.3. Data Extraction

Data were extracted with a standardized form, and counter-verified for consistency and integrity by two of the authors (AH and SBP) independently. Disagreements and discrepancies were discussed between the authors until a common accord was reached. The data collected in the standardized form included: the type of room used, the population studied, the setting (e.g., forensic, acute care psychiatric units), the main outcomes identified, the type of items or elements provided in the room (if provided), and specific architectural characteristics if provided. Elements were extracted and collected in Microsoft Excel version 2021.

### 2.4. Data Management and Analysis

Descriptive analysis of the data amassed was conducted by the authors. Compilation of the type of rooms as well as the main outcomes studied in the identified study was conducted in Microsoft Excel.

## 3. Results

### 3.1. Description of Studies

**Figure 1:**
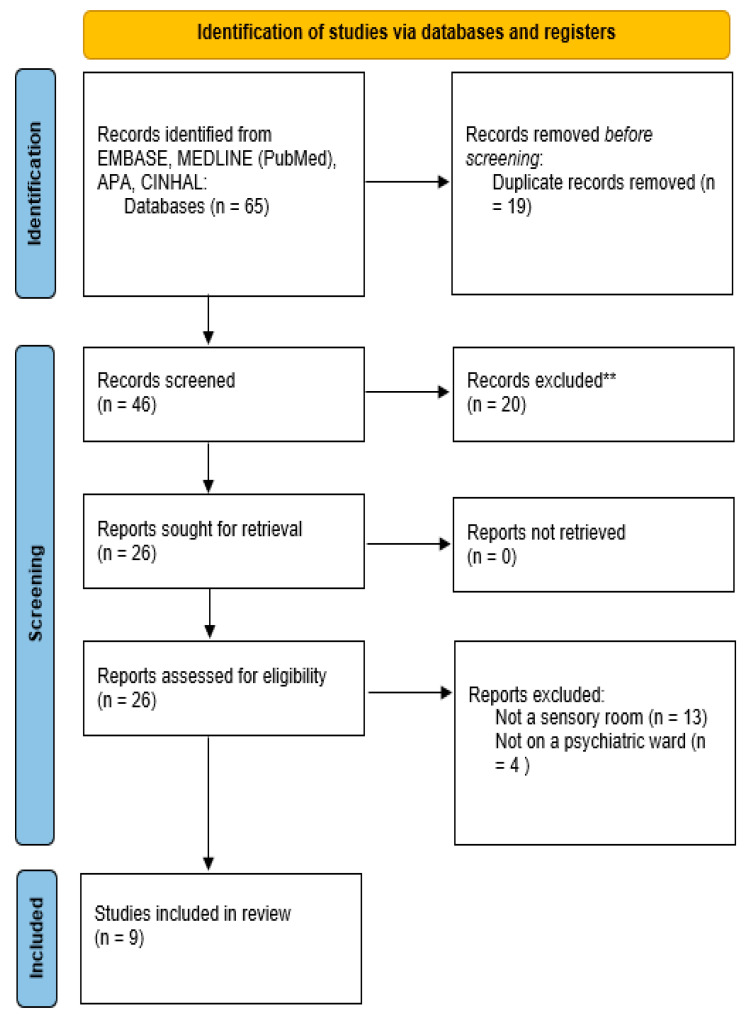
PRISMA Flowchart for the inclusion of studies. ** Records that were excluded as part of the abstract screening process.

Our scoping review assessed studies that used alternative rooms for inpatients of psychiatric units. This literature search identified 65 studies (EMBASE: N = 10, MEDLINE: N = 48, APA: N = 4, CINHAL: N = 3) that were eligible for our article from which 19 duplicates were eliminated. A total of 20 studies were excluded following abstract screening. The remaining 26 studies were assessed in their entirety of which 13 were excluded as it was found that the focus of these articles was not about an alternative room, and four were excluded as alternative rooms were not part of psychiatric units. The remaining nine studies were in fact analyzed. The PRISMA flowchart for the inclusion of studies is shown in Figure 1. It was found that solely a small number of studies engage with the specific uses for alternative rooms and that their names and modalities are very heterogenous. The studies identified and their details are presented in Table 1. Most of the studies assessed used alternative rooms to help patients in decreasing their emotional distress. Two studies reported that the use of such rooms did not reduce patients’ seclusion rates.

### 3.2. Alternative Rooms

Of the nine studies identified, six studies used the name (terminology) sensory rooms, whereas two used the terminology multisensory room (one used the term Snoezelen) and the remaining study employed the name comfort room. In Chalmers et al.’s study, which included a literature review on sensory modulation and implementation of a sensory room, they reported that several studies demonstrated that sensory approaches can be beneficial, especially when considering that symptom management alternatives for consumers experiencing distress within an inpatient psychiatric setting are lacking [23]. This statement is reported in the nine studies as sensory modulation is shown to be beneficial for many different aspects as discussed in the following section. In studies by Smith et al., Chalmers et al. and Wiglesworth et Farnworth, the perception of the staff has been considered as part of these studies whereas all the other identified studies focus solely on the patients’ reported symptoms [23,29,30].

### 3.3. The Main Uses of Alternative Rooms and Outcomes

**Figure 2:**
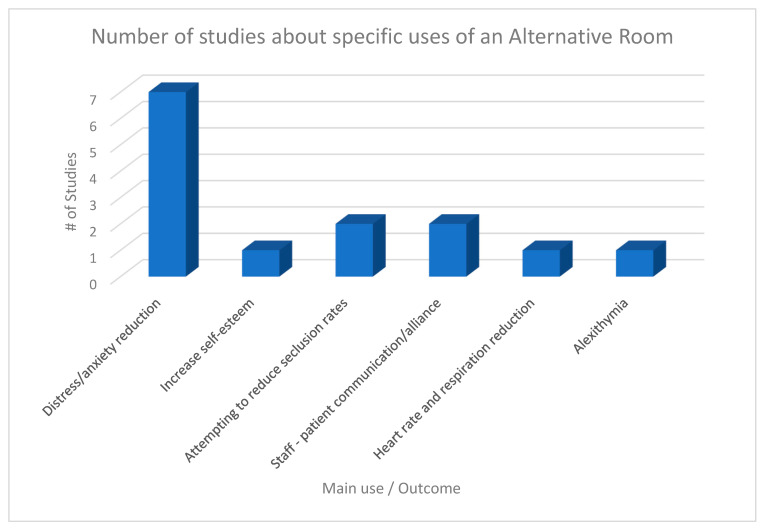
Number of studies that assessed or reported a specific use of an alternative room.

The main uses and outcomes of Alternative Rooms that were found in the identified studies are presented in Figure 2.

#### 3.3.1. Distress and Anxiety Reduction

A total of seven studies reported reductions in distress and anxiety. In Chalmers et al., pre-and post-usage of a sensory room demonstrated a significant reduction in acute arousal ratings in patients [23]. The study conducted by Dorn et al., which included 20 patients in locked rehabilitation psychiatric units, described a significant and constant decrease in arousal state as a benefit of using a sensory room [26]. Similarly, in Cheng et al., patients suffering from chronic schizophrenia self-reported a means decrease in hospital anxiety and anxiety [24]. The qualitative study realized by Hedlund Lindberg et al., a study that included 28 patients from 10 different psychiatric wards, reported a significant decrease in anxiety and an increased sense of well-being for patients [17]. Wiglesworth et Farnworth gathered focus groups which included patients of a female inpatient forensic psychiatric rehabilitation ward and staff. Both groups have shown a means decrease in overall distress as a positive consequence of using the sensory room provided by the facility [30]. Finally, Forsyth et Trevarrow, who performed a qualitative study with six health workers in an acute psychiatric ward, described a reduction in patients’ distress which was perceived by the staff [27].

#### 3.3.2. Increase of Self-Esteem

A qualitative study, conducted by Hedlund Lindberg et al. on the experiences of 28 patients in using sensory rooms in psychiatric inpatient care, reported that most participants described enhancement in self-esteem [17].

#### 3.3.3. Impact on Seclusion Rates

Two studies identified the use of alternative rooms and their correlation with seclusion rates on inpatient psychiatric units. The first identified study, conducted by Novak et al. attempted to examine the reported 54% reduction in seclusion that was mentioned in a previous study realized by Champagne et Stromberg [28,31]. They assessed 75 patients on an acute psychiatric ward setting and observed no significant changes in rates of seclusion or aggression. In a study by Smith et al., which analyzed data collected from 10 staff members and seven patients, the authors assessed if pre-and post-sensory room introduction increased or decreased since its introduction [29]. They reported that the sensory rooms did not reduce the overall rates of seclusion with the justification that solely a minority of patients were repeatedly secluded.

#### 3.3.4. Staff and Patients’ Communication and Alliance

In Smith et al., the authors highlighted how most patients and staff reported an improvement in communication between staff and patients (also between patients and their peers) as a positive outcome of using a sensory room [29]. Similar findings were reported in Forsyth et Trevarrow, a qualitative study in which six health care workers mentioned that a sensory room had a positive impact on the staff as they attended to their own emotional needs, and it supported reflective learning during critical incident debriefing [27].

#### 3.3.5. Biometrics: Heart Rate and Respiration Rate Reduction

In Cheng et al. ’s crossover study design conducted with 60 patients suffering from chronic schizophrenia, the authors reported that after six sessions of 30-min sensory intervention, participants had stabilized respiratory and heart rates [24]. They also reported that the efficacy of the multisensory stimulation room on the reported biometrics and anxiety was correlated positively with the frequency of the intervention.

#### 3.3.6. Alexithymia

An interesting study, conducted by Di Taranto et al., assessed the use of a Snoezelen multisensory room with a 19-year-old Italian woman who was visiting her older sister that was hospitalized on an inpatient psychiatric unit ward [25]. This woman was suffering from alexithymia and clinically relevant depressive and anxious symptoms. Self-reported scales demonstrated an improvement in her alexithymia and emotional openness.

## 4. Discussion

### 4.1. Review of Findings

This scoping review aimed to identify the main uses and outcomes of alternative rooms in the context of adult inpatient regular, acute, and forensic psychiatric units. A total of nine studies were identified and reported the following uses and outcomes: distress and anxiety reduction, increase in self-esteem, impact on seclusion rates, patient–staff communication and alliances, heart rate, respiration rate reduction and improvement of alexithymia. The nomenclature used to name alternative rooms varies in the identified literature using names such as sensory room, multisensory room, Snoezelen, and comfort room.

Distress and anxiety reduction has been widely reported across the identified studies. Sensory modulation contributed as a potential avenue for emotional regulation. As an example, a recent study by Adams-Leask et al., has shown a statistically significant reduction in self-reported distress in a population of 74 consumers with mental health presentations that used one to six sensory items for a median duration of 45 min [32]. Similarly, therapeutic approaches using this concept have been tested for subjects suffering specifically from anxiety. In a study by Feinstein et al., the authors conducted a within-subject crossover design of 31 participants with high anxiety sensitivity by exposing them to a 90-min session of Floatation-rest (Reduced Environmental Stimulation Therapy) and this exposition had a significant anxiolytic effect across the participants [33]. External stimuli have therefore a role in emotional distress regulation and anxiety reduction. The heart rate and respiration rate reduction identified in a study by Cheng et al. appear to be directly correlated to the reduction in anxiety as it is widely known that anxiety increases these metrics since it represents an autonomous response of the body [24,34].

The observed improvement in self-esteem could be linked to the improvement in patients’ quality of life in the inpatient units as there is literature that portrays this correlation. A cross-sectional web survey of 519 elderly people, conducted by Souza Júnior et al. (2022), recently demonstrated that self-esteem was associated with all quality-of-life facets which included sensory skills [35]. Considering that sensory skills are an important part of quality of life, it might be hypothesized that an alternative room can help in providing such facet to inpatients of psychiatric units by allowing them to connect with their sensory skills as they are hospitalized in a foreign environment. Recent findings, by Fernandes et al. (2022), highlighted that amongst 126 young adults, those who self-reported higher state of anxiety and emotional regulation issues, correlated with a state of low self-esteem [36]. A larger cross-sectional study of 6057 Chinese individuals identified a correlation between childhood trauma and self-esteem, recommending that emotional expression training as a potential avenue to reduce the negative effects of childhood trauma [37]. Since childhood trauma is more frequent in the population of patients suffering from severe mental disorders than the general population, sensory approaches such as alternative rooms might help patients with their self-esteem as it is a form of voluntary emotional expression training [38].

Alternative rooms have been reported as having no significant impact on seclusion rates in the identified studies as compared to previous studies on the subject. For example, a recent case-control study in Denmark comparing two similar psychiatric units, where one unit implemented sensory modulation as a method to prevent seclusion and the other did not, identified that the use of belts decreased by 38% as compared to the control group and the use of forced medication decreased by 46% [39]. This difference might be explained by the timing and the way that patients are exposed to the sensory room. Staff training and programs around the use of sensory modulation are included in the reported literature that indicates a decrease in seclusion rates. This might be because the sensory modulation is used to prevent distress in these studies rather than a way to help a patient going through emotional dysregulation. Nevertheless, further studies should be conducted in this area. Challenging humane behaviors is a reality on psychiatric inpatient units and tools are being developed to psychometrically assess these behaviors [40,41]. The use of alternative room combined with such instruments might be an avenue in reducing seclusion rates.

Staff and patients’ communication and alliance has been reported as improved in the two identified studies. A systematic literature review on the outcomes of sensory stimuli in critical care environment analyzed 30 articles and concluded that the physical environment can impact the patient and staff outcomes [40]. Therefore, a stimulating, personalized environment such as an alternative room, can have an impact of adding a pleasant environment for the patient, helping with their emotional regulation, and thus aiding in staff–patient communication. Similarly, the use of an alternative room to help patients suffering from alexithymia and increase patients’ openness could be related to the external stimuli presented in such a room and the patients’ sensory processing. Various sensory processing and expression difficulties are found in psychiatric disorders and must be considered as a non-specific transdiagnostic phenotype when assessing patients and designing treatment [42]. The literature describes different profiles of alexithymia, and these profiles react differently to sensory processing in the way they respond to environmental and body-based cues [43]. A stimulating environment could therefore help with sensory processing in these patients.

### 4.2. Limitations

This scoping review focused solely on the different uses of such rooms. Considering the various appellations being used and their heterogeneity, some studies might have been overviewed. Furthermore, there is a small number of studies that published information on such rooms for adult psychiatric patients which limits the amount of information that can be derived.

## 5. Conclusions

Alternative rooms have many uses for patients and staff in the context of adult inpatients psychiatric units. Hospitalized patients in psychiatric institutions may struggle with emotional control, stress management, and interpersonal problems with other patients and the staff. The nine studies that were identified in this scoping review reported the following various outcomes: distress and anxiety reduction, increase in self-esteem, impact on seclusion rates, patient–staff communication and alliances, heart rate, respiration rate reduction and improvement of alexithymia. Reducing emotional distress in patients, among the different uses identified, appears to be the predominant benefit of implementing such rooms. Sensory rooms, multisensory rooms and comfort rooms were used amongst the different appellation of such rooms. Considering the heterogeneity in the nomenclature for sensory-based rooms, and the alternate nature of such rooms as per current strategies regarding various behavioral and mental conditions, we recommend the name alternative room be used. This is to encompass all rooms in psychiatric units that have a single or several sensory components as they use various components to offer a voluntary avenue for the patients to address several difficulties such as emotional regulation. The limited amount of literature identified and the potential impact on improving patient outcomes and the quality of care in psychiatric units as identified in this review opens the door to future studies to better understand the efficacy of such rooms. Future studies are therefore needed to assess the modalities of these rooms and correlate with the identified clinical benefits. Areas of improvement in the use of alternative rooms could include research in the optimal implementation strategies of such rooms, long-term outcomes, and the impact on different patient populations.

## Figures and Tables

**Table 1:** Scoping review study selection detailed results.

Studies	Type of Room	Population (n)	Clinical Setting	Main Use and Outcomes	Type of Items	Architectural Characteristics
(Chalmers et al., 2012) [23]	Sensory room	Staff and Patients (n: N/A; N/A)	Regular psychiatric unit	Significative decrease in distress Psychometric instruments used: -Fremantle Acute Arousal Scale	Weighted items, Visual items, Olfactive items, Tactile items, Gustatory items, Auditory props	N/A
(Cheng et al., 2017) [24]	Multisensory intervention space	Patients with chronic schizophrenia (n = 60)	Regular psychiatric unit	Reduction of anxiety and stabilization of respiration and heart rates. Psychometric instruments used: - Hospital Anxiety and Depression Scale -Schizophrenia Patients’ Subjective Well-being	Weighted items, Visual items, Olfactive items, Tactile items, Gustatory items, Auditory props, Proprioceptive props	N/A
(Di Tarantino et al., 2022) [25]	Snoezelen multisensory room	The sister of a patient (n = 1)	Regular psychiatric unit	Alexithymia: Improved ability of narration of her own life and emotions Psychometric instruments used: -Minnesota Multiphasic Personality Inventory-2 -Brief Psychiatric Rating Scale -Hamilton Anxiety Scale Hamilton Rating Scale for Depression -Toronto Alexythimia Scale -Toronto Structured Interview for Alexithymia	Weighted items, Visual items, Olfactive items, Tactile items, Auditory props	N/A
(Dorn et al., 2020) [26]	Sensory room	Patients (n = 24)	Rehabilitation unit (locked)	Significantly and consistently decreased consumers’ arousal Psychometric instruments used: - Sensory Modulation Consumer Self Rating Tool	Weighted items, Visual items, Olfactive items, Tactile items, Gustatory items, Auditory props	Locked space
(Forsyth et Trevarrow, 2018) [27]	Sensory room: Chillout room	Staff (n = 6)	Acute psychiatric ward	Reducing distress. Enhancing de-escalation interventions, sensory interventions, and positive impact on staff.	Weighted items, Visual items, Tactile items, Auditory props	N/A
(Hedlund Lindberg et al., 2019) [17]	Sensory room	Patients (n = 28)	Ten regular psychiatric wards	Enhanced well-being, reduced anxiety, increased self-management and enhanced self-esteem	Weighted items, Visual items, Olfactive items, Tactile items, Gustatory items, Auditory props	N/A
(Novak et al., 2012) [28]	Comfort room	Patients (n = 75)	Acute psychiatric ward	Reduction in distress, improvements in a range of disturbed behaviors. Weighted blanket: significantly greater reduction in distress and clinician-rated anxiety. No reduction in seclusion rates. Psychometric instruments used: - Sensory room assessment form (locally developed)	Weighted items, Visual items, Olfactive items, Tactile items, Auditory props	N/A
(Smith et al., 2014) [29]	Sensory room	Staff and patients (n = 10; 7)	Intensive psychiatric care unit	Improvement in staff-patient communication. Perception of less seclusion rate by the staff but no actual reduction in seclusion rates.	Visual items, Olfactive items, Tactile items, Gustatory items, Auditory props	5 m per 2.5 m, blue painted walls, laminate flooring and one window.
(Wiglesworth et Farnworth, 2016) [30]	Sensory room	Staff and patients (n = N/A; 5)	Forensic psychiatric unit	Mean decrease in distress. Psychometric instruments used: - Adult/Adolescent Sensory Profile	Weighted items, Visual items, Olfactive items, Tactile items, Gustatory items, Auditory props	N/A

## Data Availability

Not applicable.

## Appendix A

**Table A1:** Electronic search strategy for the systematic review conducted.

	Alternative Rooms	Psychiatric Unit
EMBASE	X	psychiatric department/OR mental hospital/
MEDLINE	X	Emergency Services, Psychiatric/or Hospitals, Psychiatric/or Psychiatric Department, Hospital/
APA	sensory room/comfort room	psychiatric hospitals/or psychiatric units/or mental health clinics/
CINHAL	X	
Open vocabulary	(((Room OR space OR centre OR studio) N3 (Sport OR sports OR fitness OR relaxation OR meditation OR game OR games OR gaming OR boardgame OR boardgames OR play OR yoga OR alternative OR creative OR art OR arts OR ludic OR multisensory OR sensory OR dining OR quiet or well-being OR well-being OR leisure OR recreation OR recreational OR recreative OR music OR alternative)) OR (Gym OR theater OR swimming pool OR garden OR library))	(psychiatric OR mental) N2 (department* OR Unit * OR ward * OR hospital * OR clinic * OR centre*)

Search strategy: (sensory room/OR (((Room OR space OR centre OR studio) ADJ3 (Sport OR sports OR fitness OR relaxation OR meditation OR game OR games OR gaming OR boardgame OR boardgames OR play OR yoga OR alternative OR creative OR art OR arts OR ludic OR multisensory OR sensory OR dining OR quiet or well-being OR well-being OR leisure OR recreation OR recreational OR recreative OR music OR alternative)) OR (Gym OR theater OR swimming pool OR garden OR library)).tw.) AND (((psychiatric OR mental) ADJ2 (department * OR Unit * OR ward * OR hospital * OR clinic * OR centre *)).tw. OR (psychiatric hospitals/or psychiatric units/or mental health clinics/)).
